# Soluble epoxide hydrolase inhibitors, t-AUCB, regulated microRNA-1 and its target genes in myocardial infarction mice

**DOI:** 10.18632/oncotarget.21831

**Published:** 2017-09-18

**Authors:** Ya-Jun Gui, Tao Yang, Qiong Liu, Cai-Xiu Liao, Jing-Yuan Chen, Ya-Ting Wang, Jia-Hui Hu, Dan-Yan Xu

**Affiliations:** ^1^ Department of Cardiology, Internal Medicine, The Second Xiangya Hospital, Central South University, Changsha, Hunan 410011, China; ^2^ Department of Cardiology, Internal Medicine, Changsha Central Hospital, Changsha, Hunan 410011, China; ^3^ Department of Geratology, Internal Medicine, The Third Hospital of Changsha, Changsha, Hunan 410011, China

**Keywords:** soluble epoxide hydrolase inhibitors, miR-1, ischemic arrhythmia

## Abstract

**Purpose:**

Soluble epoxide hydrolase inhibitors (sEHIs) had been demonstrated to produce cardioprotective effects against ischemia-induced lethal arrhythmias, but the exact mechanisms remain unknown. The present study was designed to investigate whether the beneficial effects of sEHIs are related to regulation of microRNA-1, which was a proarrhythmic factor in the ischemic heart.

**Methods:**

A mousemyocardial infarction (MI) model was established by ligating the coronary artery. sEHI t-AUCB (0.2, 1, 5 mg/L in drinking-water) was administered daily seven days before MI. The incidence of arrhythmias was assessed by *in vivo* electrophysiologic studies. miR-1, *KCNJ2* (encoding the K^+^ channel subunit Kir2.1), and *GJA1* (encoding connexin 43 [Cx43]) mRNA were measured by real-time PCR; Kir2.1 and Cx43 protein were assessed by western blotting and immunohistochemistry.

**Results:**

We demonstrated that sEHIs reduced the myocardium infarct size and incidence of inducible arrhythmias in MI mice. Up-regulation of miR-1 and down-regulation of *KCNJ2*/Kir2.1 and *GJA1*/Cx43 mRNA/protein were observed in ischemic myocaridum, whereas administration of sEHIs produced an opposite effect. In addition, miR-1 overexpression inhibited expression of the target mRNA and their corresponding proteins, whereas t-AUCB reversed the effects. Our results further revealed that PI3K/Akt signaling pathway might participate in the negatively regulation of miR-1 by sEHi.

**Conclusions:**

We conclude that sEHIs can repress miR-1, thus stimulate expression of *KCNJ2*/Kir2.1 and *GJA1*/Cx43 mRNA/protein in MI mice, suggesting a possible mechanism for its potential therapeutic application in ischemic arrhythmias.

## INTRODUCTION

Life-threatening ischemic arrhythmias caused by myocardial infarction (MI) appear to be the leading cause of sudden cardiac death. As conventional treatment relies on the classic anti-arrhythmic drugs, which have limited efficiency and pro-arrhythmic potential, it is crucial to discover more effective drugs against lethal ischemic arrhythmias.

Epoxyeicosatrienoic acids (EETs) are endogenous oxylipids and have been shown to produce a wide variety of cardioprotective effects, such as causing marked vasodilation, inhibiting platelet aggregation and adhesion, and modulating lipid metabolism [1–3]. Most EETs are unstable and convert rapidly into biologically inactive product dihydroxyeicosatrienoic acids (DHETs) by soluble epoxide hydrolase (sEH) enzyme. The sEH inhibitors (sEHIs) can enhance the beneficial effects of EETs by increasing the level of endogenous EETs.

Several studies have documented the cardioprotective effects of sEHIs in many different pathological conditions, including cardiac hypertrophy, heart failure, hypertension and coronary heart disease [4–9]. sEHIs also had a beneficial effect in the prevention of cardiac arrhythmias both in murine models with hypertrophy and MI [9–11]. We have previously demonstrated that sEHIs have anti-arrhythmic effects by repressing the activation of NF-κB-mediated gene transcription in a murine model with hypertrophy [9]. However, the exact mechanisms by which sEHIs exert its anti-arrhythmic effect after MI have remained largely elusive.

MicroRNAs (miRNAs) are conserved 22-nucle-otide non-coding RNAs that act as post-transcriptional repressors of target genes via antisense binding to the 3′ untranslated regions of target mRNAs, resulting in mRNA degradation and/or translational repression. Recently, microRNAs (miR) dysregulation after cardiac injury had been implicated in several biological processes involved in cardiovascular disease [12]. miR-1 had been demonstrated to be a potential arrhythmogenic factor in ischemic heart. miR-1 could downregulates *KCNJ2* mRNA, resulting in decreased Kir2.1 protein, the K^+^ channel subunit responsible for inward rectifier K^+^ current (I_k1_). Decreased I_k1_ contributes to slowing repolarization and prolonging QT. Furthermore, miR-1 could also inhibit the expression of connexin 43 (Cx43) protein, resulting in slowed electrical conduction between adjacent cardiomyocytes and in strengthened early after depolarization [13]. Therefore, miR-1 might be a new target for treating lethal ischemic arrhythmias.

The aim of the present study was to investigate whether the anti-arrhythmic effects of sEHi were related to miR-1 expression in a mouse model of MI. To this end, we evaluated the effects of sEHi trans-4-[4-(3-adamantan-1-yl-Ureido)-cyclohe-xyloxy]-benzoic acid (t-AUCB) on arrhythmia incidence, and the expression of miR-1 and its target arrhythmia–related genes.

## RESULTS

### Effect of t-AUCB on infarct size

**Figure 1:**
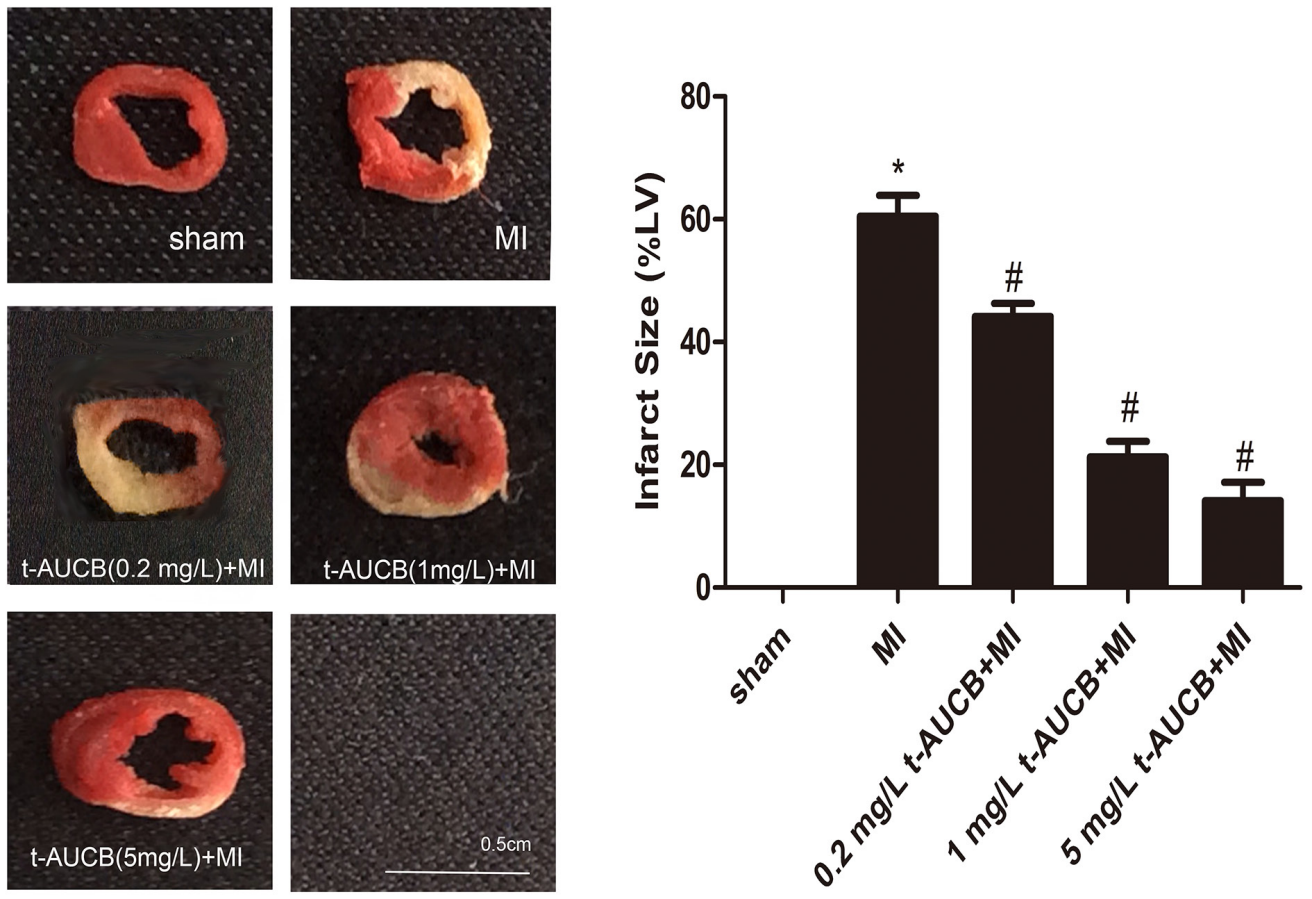
t-AUCB decreased infarct size in MI mice Representative images of 2,3,5-triphenyltetrazolium chloride (TTC) staining in t-AUCB-treated or control hearts (left). Surviving tissue stained red with TTC and infarcted tissue was white. Infarct size expressed as percentage of left ventricular area for each group (right). Bars represented mean±SEM; ^*^P<0.05 vs. sham group; #*P*<0.05 vs. MI group. n=3.

The results were shown in Figure 1. Compared with MI group, the myocardium infarct size were decreased from 62% to 45%, 21%, and 14% in MI mice treated with 0.2 mg/L, 1 mg/L, and 5 mg/L t-AUCB, respectively (all *P* < 0.05).

### Effects of t-AUCB on arrhythmias in MI mice

**Figure 2:**
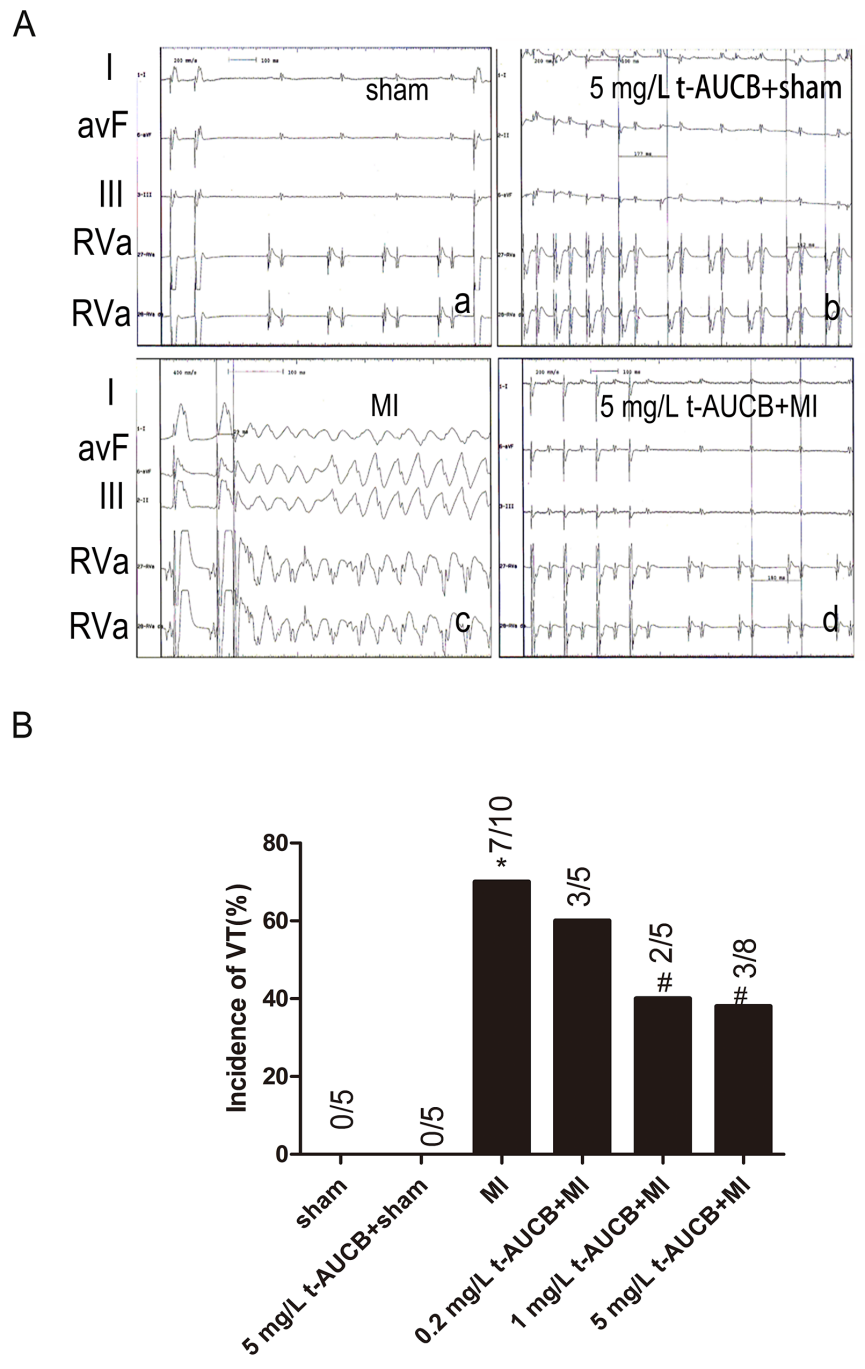
t-AUCB protected against ischemic arrhythmia inducibility in MI mice MI was established by coronary occlusion for 24 h. **(A)** Examples of surface electrocardiogram and simultaneous intracardiac electrograms from atria and ventricles from sham-operated or MI mice treated with or without t-AUCB (5mg/L). Upper three tracing were surface ECG (Lead I, aVF, III). Lower two tracings were intracardiac electrogram showing atrial and ventricular electrograms. Summary data for the incidence of inducible ventricular tachycardia were shown in **(B)**. ^*^P<0.05 vs. sham group; #*P* <0.05 vs. MI group. n=5-10 for each group.

We performed *in vivo* electrophysiologic studies (EPS) to test whether sEHIs have salutary effects on ischemic arrhythmias in the setting of MI. Shown in Figure 2A were examples of surface electrocardiogram and simultaneous intracardiac electrograms from atria and ventricles from sham-operated or MI mice treated with or without t-AUCB (5 mg/L). *In vivo* EPS in untreated MI mice showing evidence of inducible ventricular tachycardia (VT) (Figure 2c). However, MI mice treated with t-AUCB decreased the incidence of inducible VT (Figure 2d). Summary data for the incidence of VT were shown in Table 1 and Figure 2B. Figure 2B illustrated that the susceptibility to arrhythmia of the MI mice at baseline and after pretreatment with t-AUCB (0.2, 1 and 5 mg/L). At baseline, 7 of 10 MI mice (70%) had inducible ventricular tachycardia (VT) during programmed stimulation. Compared with the MI group, the incidence of VT decreased to 40%, and 38% in MI mice treated with 1 mg/L and 5 mg/L t-AUCB (all *P* < 0.05), respectively. The susceptibility to increased ventricular arrhythmias was significantly suppressed in MI mice treated with sEHIs. In contrast, transfection of miR-1 agomir promoted ischemic arrhythmias. However, co-application of t-AUCB and miR-1 agomir could rescue this effect. No spontaneous arrhythmias were observed in sham-operated mice. Summary data were shown in Supplementary Table 1.

**Table 1:** Arrhythmia Vulnerability in mice treated with t-AUCB

Groups	VT	AF
sham (n=5)	0	0
t-AUCB(5 mg/L)+ sham (n=5)	0	0
MI (n=10)	7(70%)^*^	3(30%)^*^
t-AUCB(0.2 mg/L) + MI (n=5)	3(60%)	1(20%)
t-AUCB(1 mg/L) + MI (n=5)	2(40%)^#^	1(20%)
t-AUCB(5 mg/L) + MI (n=8)	3(38%)^#^	2(25%)

VT, ventricular tachycardia; AF, atrial fibrillation. ^*^P<0.05 vs. sham group; #P<0.05 vs. MI group.

### Effects of t-AUCB on levels of miR-1, KCNJ2 and GJA1 mRNA in MI mice

**Figure 3:**
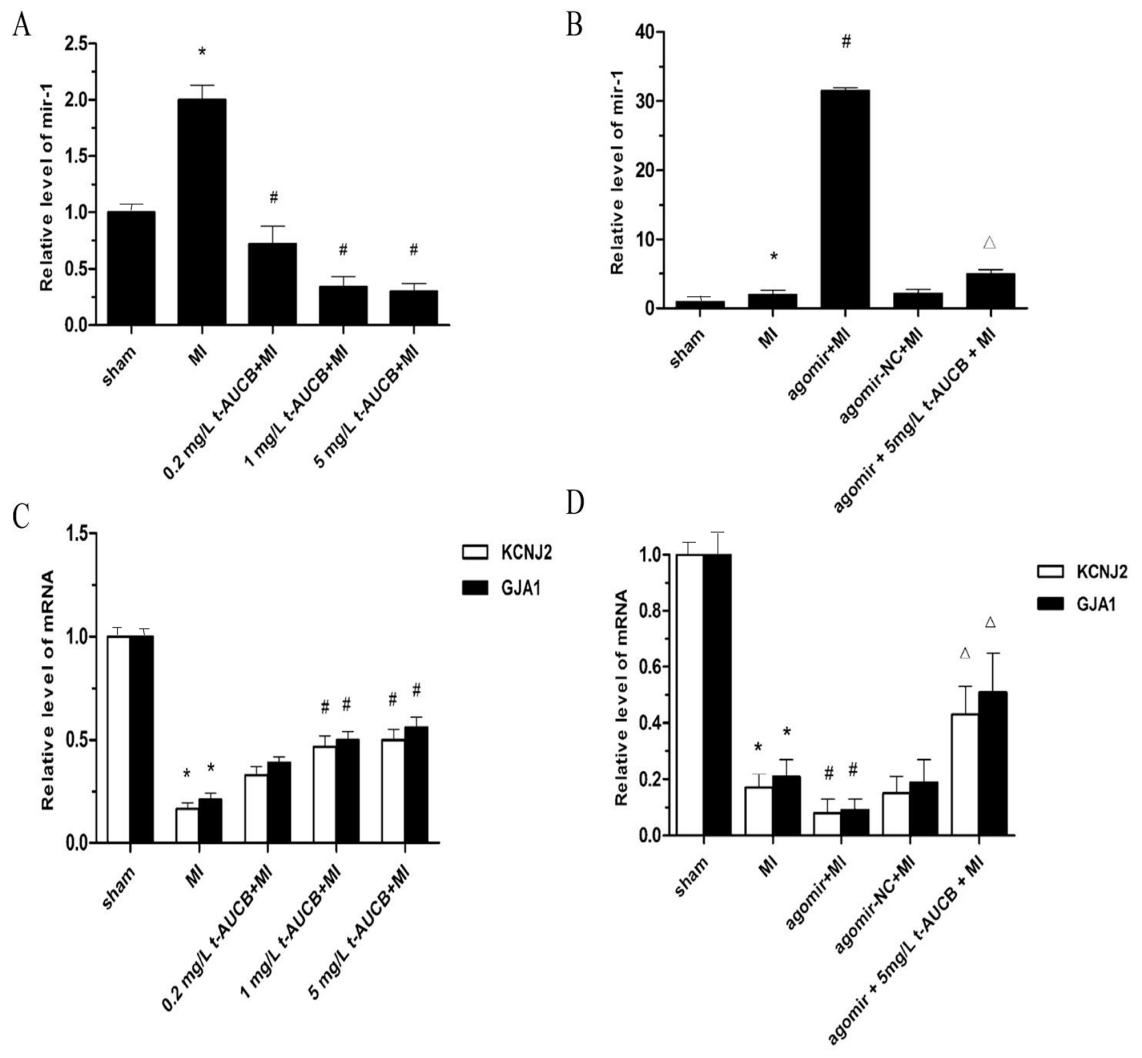
t-AUCB prevented upregulation of miR-1 and restored the expression of *KCNJ2* and *GJA1* mRNA in ischemic myocardium **(A)** Ischemic upregulated miR-1 expression in MI hearts, while t-AUCB suppressed miR-1 expression in a dose-dependent manner. miR-1 level were quantificated by real-time PCR with RNA samples isolated from mice hearts 24 h after MI. **(B)** The upregulation of miR-1 was exacerbated by agomir in MI hearts, but alleviated by t-AUCB. **(C)** Ischemic downregulated *KCNJ2* and *GJA1* mRNA expression in MI hearts, while t-AUCB restored *KCNJ2* and *GJA1* mRNA expression in a dose-dependent manner. **(D)** Levels of both *KCNJ2* and *GJA1* mRNA expression were reduced in MI and the reduction was exacerbated by agomir, but alleviated by t-AUCB. Data were expressed as mean ± SEM; ^*^P<0.05 vs. sham group; #*P*<0.05 vs. MI group; Δ*P*<0.05 vs agomir +MI group, n=7.

As shown in Figure 3A, miR-1 level in the ischemic myocardium of the MI group were increased by 2.02-fold as compared with the sham group (*P* < 0.05). t-AUCB suppressed miR-1 expression dose-dependently. Compared with the MI group, miR-1 level were decreased to 36%, 17%, and 10% in MI mice treated with 0.2 mg/L, 1 mg/L, and 5 mg/L t-AUCB, respectively (all *P* < 0.05).

We injection the agonist miR-1 agomir (10 nM) via the tail vein and found that agomir treatment caused a 16.07-fold increase in miR-1 level in the MI mice (*P* < 0.05). This increased tendency of miR-1 was abolished by pretreatment with t-AUCB. miR-1 level were decreased to 16% in the agomir+5 mg/L t-AUCB+MI group as compared to the agomir+MI group (*P* < 0.05, Figure 3B). In addition, we also tested the distribution of miR-1 agomir after *in vivo* transfer procedures (Supplementary Figure 3). They were mostly distributed within the area of the ischemic zone.

Because *KCNJ2* (encodes Kir2.1) and *GJA1* (encodes Cx43) were targets of miR-1, we further investigated of the effects of t-AUCB on the expression of *KCNJ2* and *GJA1* mRNA. As illustrated in Figure 3C, *KCNJ2* and *GJA1*mRNA level were decreased to 17% and 21%, respectively, in the ischemic myocardium of the MI group as compared with the sham group (all *P* < 0.05). t-AUCB upregulated *KCNJ2* and *GJA1*mRNA expression in dose-dependently. Compared with the MI group, *KCNJ2* mRNA expression was increased 2.36-fold and 2.63-fold in MI mice treated with 1 mg/L and 5 mg/L t-AUCB, respectively (all *P* < 0.05). Likewise, there were 2.75-fold and 3.01-fold increases in *GJA1*mRNA expression in MI mice treated with 1 mg/L and 5 mg/L t-AUCB, respectively (all *P* < 0.05).

We used the agomir to further investigate the link between miR-1, *KCNJ2* and *GJA1* mRNA, and t-AUCB. *KCNJ2* and *GJA1*mRNA level were significantly decreased in the hearts of MI mice as compared with the sham mice (all *P*< 0.05). *KCNJ2* and *GJA1*mRNA level were decreased to 54% and 50%, respectively, in the agomir+MI group as compared to the MI group (all *P* < 0.05). This reduction was reversed by the 5 mg/L t-AUCB pretreatment, which caused a 5.3-fold and 5.67-fold increase in *KCNJ2* and *GJA1* mRNA expression, respectively (all *P* < 0.05, Figure 3D).

### Effects of t-AUCB on Kir2.1 and Cx43 protein in MI mice

**Figure 4:**
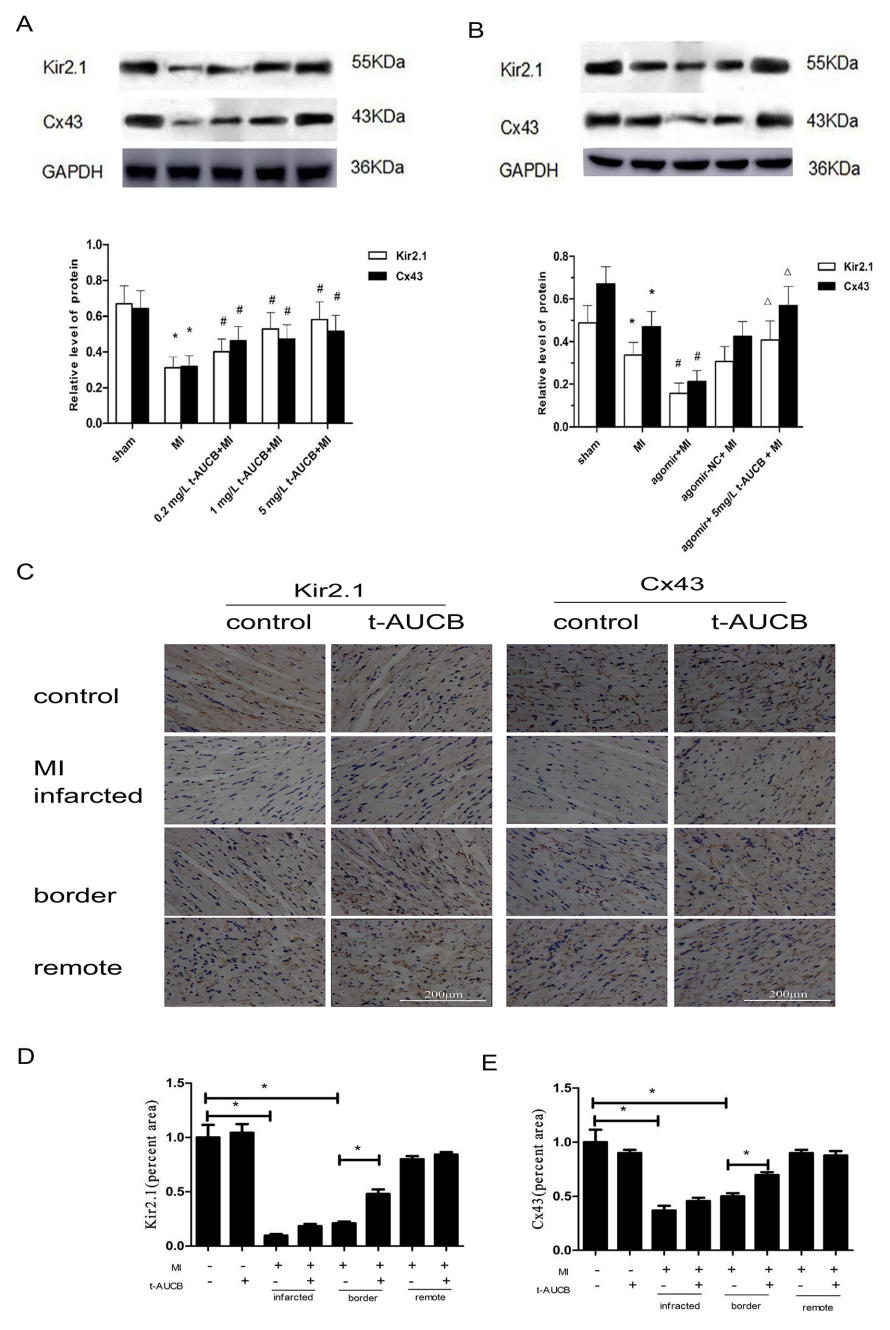
t-AUCB restored the expression of *KCNJ2* and *GJA1* at the protein level (Kir2.1 and Cx43, respectively) in ischemic myocardium **(A)** Ischemic downregulated Kir2.1 and Cx43 protein expression in MI hearts, while t-AUCB restored Kir2.1 and Cx43 protein expression in a dose-dependent manner. Measurements were made 24 h after MI. Top, examples of western blot bands; bottom, quantitation as mean ± SEM. **(B)** Levels of both Kir2.1 and Cx43 were reduced in MI and the reduction was exacerbated by agomir, but alleviated by t-AUCB. Top, examples of western blot bands; bottom, quantitation as mean ± SEM. ^*^P<0.05 vs. sham group; #*P*<0.05 vs. MI group; Δ*P*<0.05 vs agomir +MI group, n=7. **(C)** Representative immunohistochemical staining of myocardium probed with anti-Kir2.1 and anti-Cx43 antibody. Cx43 was distributed at the intercalated discs between cardiomyocytes in myocardium. **(D)** Semiquantitative analysis of Kir2.1 (percent area). **(E)** Semiquantitative analysis of Cx43 (percent area). ^*^P<0.05, n=3.

We determined the effects of t-AUCB on the expression of the protein products of *KCNJ2* and *GJA1* mRNA (Kir2.1 and Cx43, respectively) by western blotting. As shown in Figure 4A, Kir2.1 and Cx43 protein were decreased to 47% and 50%, respectively, in MI mice as compared with sham mice (all *P* < 0.05). These changes were reversed by t-AUCB treatment. Compared with the MI group, Kir2.1 protein expression increased 1.28-fold, 1.69-fold, and 2.0-fold in MI mice treated with 0.2 mg/L, 1 mg/L, and 5 mg/L t-AUCB, respectively (all *P* < 0.05). Likewise, the Cx43 protein level in MI mice treated with 0.2 mg/L, 1 mg/L, and 5 mg/L t-AUCB increased dose-dependently by 1.44-fold, 1.69-fold, and 1.85-fold, respectively (all *P* < 0.05).

We then investigated the link between miR-1, Kir2.1 and Cx43, and t-AUCB by *in vivo* gene transfer. Kir2.1 and Cx43 protein level were decreased to 69% and 70%, respectively, in MI mice as compared with the sham mice (all *P* < 0.05). Compared with the MI group, Kir2.1 and Cx43 protein expression was decreased to 46% and 45%, respectively, after transfected of miR-1 agomir (all *P* < 0.05). This reduction was reversed by the 5 mg/L t-AUCB pretreatment, which caused a 2.62-fold and 2.66-fold increase in Kir2.1 and Cx43 protein expression, respectively (all *P* < 0.05, Figure 4B).

Immunochemistry analysis showed the same tendency (Figure 4C–4E). The expression of Kir2.1 and Cx43 was low in the border zone of the myocardium in MI group, but t-AUCB could increase them. However, t-AUCB showed no effect on the expression of Kir2.1 and Cx43 in sham group.

### Potential role of AKT/GSK3β signaling pathway in miR-1 reduction by sEHi

**Figure 5:**
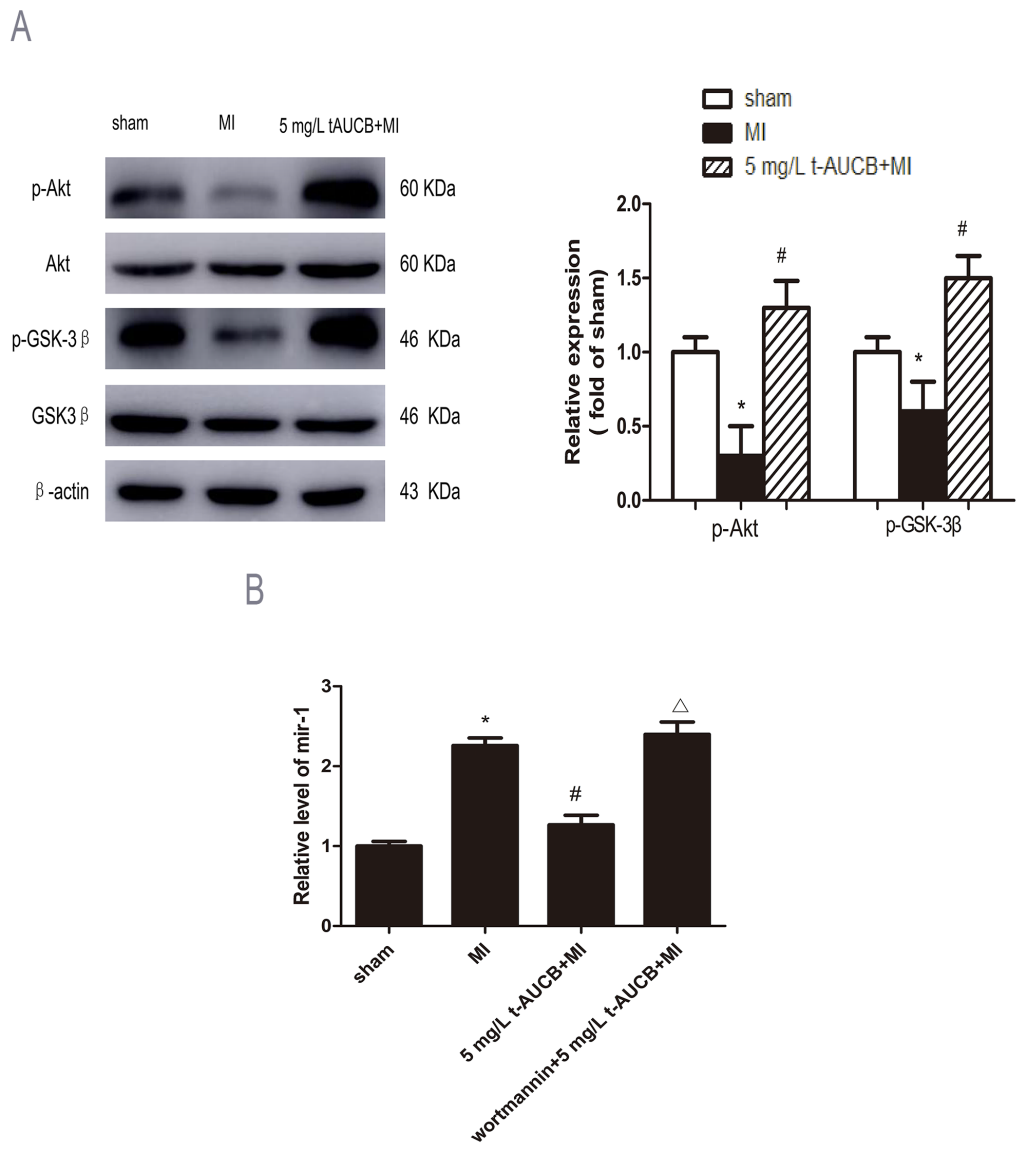
AKT/GSK3β signaling pathway participated in regulation of miR-1 by sEHi **(A)** Ischemic downregulated PKA and GSK3β expression in MI hearts, while 5 mg/L t-AUCB restored PKA and GSK3β expression. Measurements were made 24 h after MI. Left, examples of western blot bands; Right, relative expression level of p-Akt and p-GSK3β ratio to total AKT and GSK3β, respectively. Quantitation as mean ± SEM. ^*^P<0.05 vs. sham group; #*P*<0.05 vs. MI group; n=3. **(B)** Levels of miR-1 was reduced in MI mice treated with 5 mg/L t-AUCB, while PI3K inhibitor wortmannin suppressed the downregulation of miR-1. miR-1 level were quantificated by real-time PCR with RNA samples isolated from mice hearts 24 h after MI. Data were expressed as mean ± SEM; ^*^P<0.05 vs. sham group; #*P*<0.05 vs. MI group; Δ*P*<0.05 vs 5 mg/L t-AUCB +MI group, n=3.

The expression of AKT, p-AKT, GSK3β and p-GSK3β were analyzed in MI mice pretreatment with sEHi t-AUCB for 1 week. After normalized by total AKT, p-AKT were decreased to 30% in MI mice as compared with sham mice (*P* < 0.05). This trend was reversed by 5 mg/L t-AUCB treatment. Compared with the MI group, p-AKT protein expression was increased to 1.3-fold in MI mice treated with 5 mg/L t-AUCB (*P* < 0.05, Figure 5A).

Consistently, p-GSK3β, a downstream target of AKT, after normalized by total p-GSK3β, p-GSK3β was decreased to 60% in MI mice compared to sham mice ( *P* < 0.05). This reduction was reversed by t-AUCB pretreatment, which caused a 1.5-fold increase in p-GSK3β expression (*P* < 0.05, Figure 5A).

To confirm that AKT/GSK3β pathway was involved in regulation of miR-1, we injected t-AUCB-treated MI mice with PI3K inhibitor wortmannin (0.3 mg/kg) via the tail vein. We found the down-regulation of miR-1 by 5 mg/L t-AUCB was abolished by pretreatment with wortmannin, which caused a 2.1-fold increase in miR-1 level compared to the 5 mg/L t-AUCB+MI group (*P*<0.05, Figure 5B)

### Effects of t-AUCB treatment on EETs concentration

**Figure 6:**
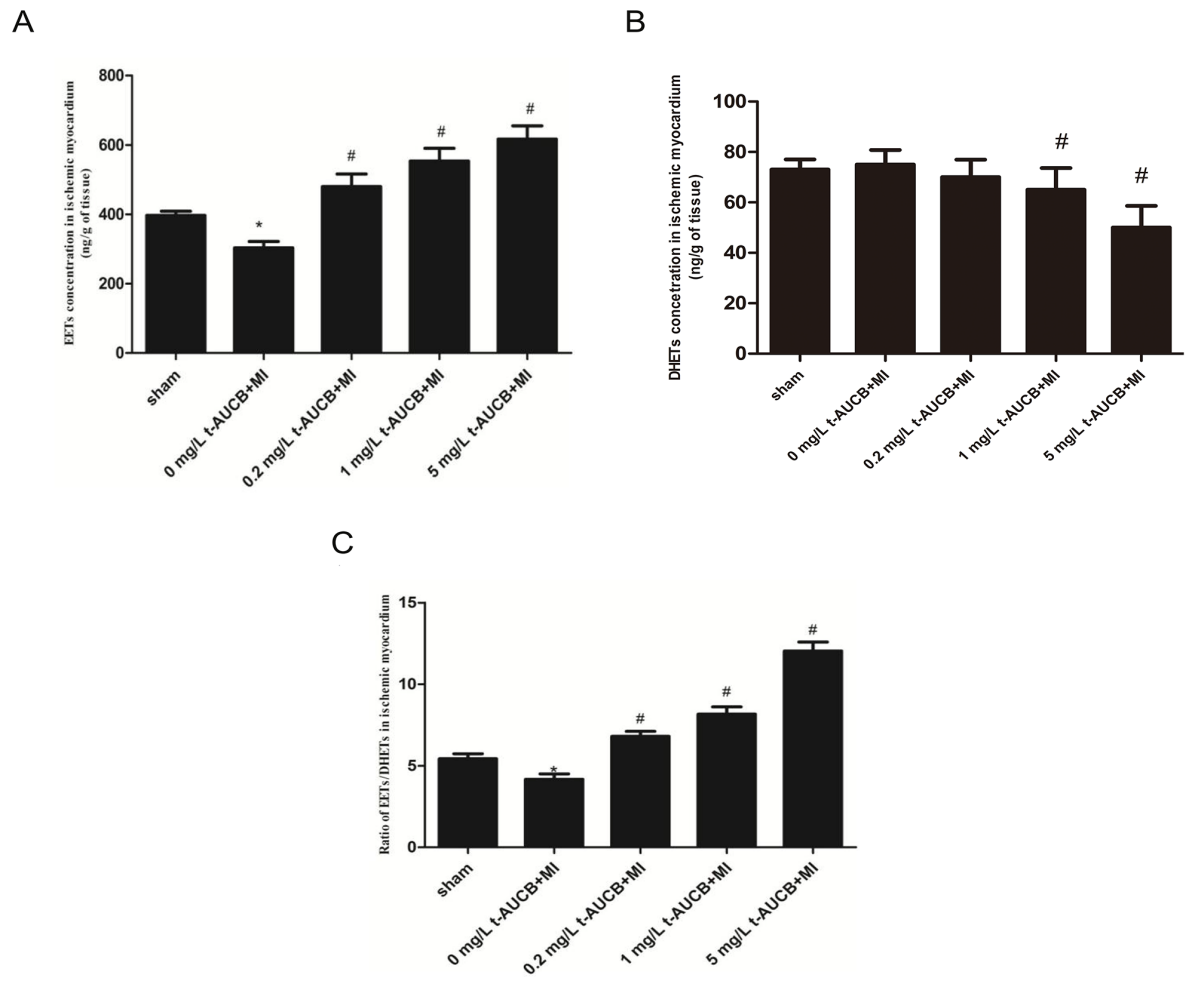
Epoxyeicosatrienoic acids (EETs) **(A)** and dihydroxyeicosatrienoic acids. (DHETs) **(B)** in ischemic myocardium of MI mice and the ratio of EETs to DHETs **(C)**, as an indicator of t-AUCB treatment efficiency. Before the MI surgery or sham-operated, mice were randomized to receive either drinking-water or t-AUCB (0.2, 1, 5mg/L) for seven days. Measurements were made 24h after MI. The concentration of EETs and DHETs in the ischemic myocardium was measured by reverse-phase HPLC method (A,B), and the sEH activity in the ischemic myocardium was presented by ratio of EETs to DHETs (C). ^*^P<0.05 vs. sham group; #*P*<0.05 vs. MI group, n=7.

The concentration of EETs in the ischemic myocardium of MI mice was significantly increased in a dose-dependent manner after treatment with 0.2 mg/L, 1 mg/L and 5 mg/L of t-AUCB ( all *P*<0.05, Figure 6A). DHETs, products of the degradation of EETs, were higher in MI mice than those in t-AUCB (1 and 5 mg/L) treated MI mice (all *P*<0.05, Figure 6B). Figure 6C summarized the results on the availability of biologically active epoxygenase metabolites in the ischemic myocardium when expressed as the EETs/DHETs ratio. This ratio was significantly lower in MI mice than in sham mice (*P*<0.05). Treatment with t-AUCB (0.2, 1, 5 mg/L) significantly increased this ratio in a dose-dependently manner ( all *P*<0.05, Figure 6C).

## DISCUSSION

Our study yielded a novel finding. We demonstrated, for the first time, that sEHi t-AUCB could abolish the repressing effects of miR-1 on *KCNJ2*/Kir2.1 and *GJA1*/Cx43 mRNA/protein in MI mouse hearts. These findings not only help us understand the mechanisms underlying the anti-arrhythmic effects of sEHi but also conceptually advance our view of miRNAs as potential therapeutic and drug targets.

MI resulted in alterations in ion channel protein expression and gap junction expression and distribution, which contribute to electrical disturbance that leads to life-threatening cardiac arrhythmias. It had been established that miR-1 was a proarrhythmic factor in the MI heart. In the present study, we observed a 2.0-fold increase in the ischemic myocardium at 24 h post-MI. Consistent with our study, Shan et al. [14] reported that miR-1 level were increased 1.2-fold and 2.9-fold in rat hearts at 6 h and 3 months, respectively, after MI. However, the upregulation of miR-1 in ischemic myocardium after MI was incompletely understood. It was thought that the augmentation of β-adrenoceptor (βAR)- cyclic adenosine monophosphate (cAMP) -protein Kinase A (PKA) pathway might contribute to the upregulation of miR-1 [15]. The activation of βAR/cAMP/PKA signaling play an important role in the pathogenesis of MI. The activation of PKA could be translocated into nucleus where it can subsequently phosphorylate cAMP-responsive element binding protein CREB. Activation of transcription factor CREB was able to promote the transcription of miR-1 gene.

Up-regulation of miR-1 in MI mice might be involved in the development of life-threatening arrhythmias such as ventricular tachycardia (VT), ventricular fibrillation, and atrioventricular block (AVB) by targeting the mRNAs of ion channel genes, i.e., *KCNJ2* and *GJA1* [16, 17], respectively. *KCNJ2* encoded Kir2.1, the main potassium channel subunit carrying I_k1_, and is responsible for maintaining the cardiac resting membrane potential (RMP). Decreased I_k1_ caused by down-regulation of Kir2.1 protein couldlead to depolarized cardiac resting membrane potential and prolonged action potential duration (APD) in MI hearts. The down-regulation of I_k1_ might also predispose to QT prolongation. The dysfunction of I_k1_ could result in electrophysiological disorders and increase the risk for life-threatening arrhythmias [18, 19]. Cx43 is responsible for intercellular conductance in the ventricles, and the loss of Cx43 reduces electrical coupling, resulting in early afterdepolarizations (EAD), prolonged APD, and delayed conduction. The extreme conduction slowing might permit the occurrence of reentrant excitation in extremely small areas of cardiac tissue, namely microreentry [20]. Therefore, down-regulation of miR-1 might provide protection against ischemic arrhythmia.

The sEHIs have multiple biological functions, such as decreasing infarct size and inhibiting cardiac hypertrophy and cardiac fibrosis. More importantly, in our present study, we found that sEHIs could also reduce the incidence of ventricular arrhythmias in MI mice. Consistent with our finding, Shrestha et al. [11] reported that sEHi t-AUCB could significantly prevent elecrocardiographic (EKG) abnormalities, such as prolongation of QTc interval, ST height depression, pathological Q-wave formation in isoproterenol-induced MI rats. However, the underlying mechanisms for the anti-arrhythmia effects of sEHi still remain unknown. Growing evidence indicated that microRNA-1 (miR-1) was a proarrhythmic factor in the ischemic heart. Therefore, we explore the effect of sEHi on the expression of miR-1. In our preliminary experiments, we found that the miR-1 level in ischemic myocardium of MI mice was significantly increased in a dose-dependent manner after treatment with 5, 15 and 50 mg/L t-AUCB (Supplementary Figure 1A). However, in our present study, we observed that miR-1 level was decreased in dose-dependently in MI mice treated with 0.2, 1 and 5 mg/L t-AUCB (Figure 3A). Therefore, we could conclude that 5 mg/L might be the recommended dosage of t-AUCB to suppress miR-1 expression. The effect of low dose (less than 5 mg/L) and high dose (great than 5 mg/L) t-AUCB on miR-1 expression were different. The exact mechanisms remain unclear. It was well known that sEHIs have beneficial effects on cardiovascular system by increasing EETs production. EETs could act as peroxisome proliferator-activated receptors gamma (PPARγ) agonists and protected against electrical remodeling, VT, and atrial fibrillation susceptibility in animals with cardiac hypertrophy [21]. Wallace et al. [22] demonstrated that the PPARγ coactivator 1 (PGC-1) was capable of acviting nuclear serum response factor (SRF) protein following endurance exercise. SRF played an important role in regulating the expression of miRNAs. Overexpression of SRF led to a downregulation of many SRF-dependent miRNAs in the heart including miR-1. Therefore, we hypothesized that low dose sEHIs decreased expression of miR-1 in MI mice was mediated at least in part via up-regulation of SRF protein. In contrast, Johnson et al. [23] reported that the levels of SRF protein was inversely related in response to treatment with PPARγ agonist docosahexaenoic acid. Thus, PPARγ agonist could also decrease level of SRF protein, which might in part mediated the up-regulation of miR-1 in MI mice treated with high dose sEHi. Our further work confirmed that the effect of low dose and high dose t-AUCB on expression of SRF protein was different (Supplementary Figure 1B).

Here, we demonstrated for the first time, that the sEHi t-AUCB dose-dependently suppressed miR-1 upregulation in ischemic myocardium, which might be one of the mechanisms underlying the anti-arrhythmic effect of sEHi. Consistently, our previous *in vitro* whole-cell patch-clamp recording demonstrated that the current density of I_k1_ was markedly decreased in miR-1 overexpression model of neonatal cardiac myocytes [24]. The decreased I_k1_ was an important proarrhythmic factor in cardiac myocytes.. The reduction of I_k1_ was prevented by sEHi t-AUCB. These restults indicated that sEHi negatively regulated the expression of miR-1. However, the actual mechanism responsible for miR-1 downregulation by t-AUCB in the ischemic myocardium remains poorly understood.

The activation of βAR/cAMP/PKA signaling might contribute to the upregulation of miR-1 in the pathogenesis of MI [15]. And there were much evidence to show that EETs could activate cAMP/PKA. It was therefore expected that the sEHi might up-regulate miR-1 expression in ischaemic heart. However, our present study found that miR-1 was negatively regulated by sEHi. The divergent results might be due to the activation of PI3K/Akt pathway by sEHi, which was confirmed in our present study.

Our result showed that there were significantly increased AKT and GSK3β phosphorylation in t-AUCB treated MI mice. In line with this, Jiang et al. [25] reported that sEHi could up-regulate the expression of eNOS by activating PI3K/Akt pathway. Similarly, Dhanasekaran et al. [26] also found that sEHi could protect cardi-omyocytes from hypoxia/anoxia via activation of PI3K pathway. Our study further found that the application of PI3K inhibitor wortmannin abolished the inhibitory effect of sEHi on miR-1. It was therefore expected that sEHi t-AUCB could down-regulate miR-1 expression in ischemic heart by activating PI3K/Akt pathway.

Another important finding of this study was that the sEHi t-AUCB restored the impaired *KCNJ2*/Kir2.1 and *GJA1*/Cx43 mRNA/protein expression in ischemic myocardium via suppression miR-1. We first demonstrated that the expression of *KCNJ2* and *GJA1* mRNA were decreased in ischemic myocardial of mice, whereas t-AUCB restored *KCNJ2* and *GJA1* mRNA expression in a dose-dependent manner, which suggested a dose-effect relationship between sEHi and *KCNJ2* and *GJA1* mRNA (Figure 3C). Second, it was well known that *KCNJ2* and *GJA1* were targets of miR-1. miR-1 could target the 3’ untranslated region (3’UTR) of *KCNJ2* and *GJA1* mRNA and suppressed the expression of *KCNJ2* and *GJA1* mRNA. Therefore, *KCNJ2* and *GJA1* mRNA were direct negatively modulated by miR-1. More important, we further demonstrated that sEHi t-AUCB could restore the expression of *KCNJ2* and *GJA1* mRNA, which were repressed by the agonist miR-1 agomir (Figure 3D). The result further demonstrated that sEHi indirect effected the expression of *KCNJ2* and *GJA1* mRNA via suppression miR-1.

In the present study, we observed the obvious downregulation of *KCNJ2*/Kir2.1 and *GJA1*/Cx43 mRNA/protein induced by miR-1 overexpression when agomir were injected via the tail vein. Given our results, we could assume that the overexpression of miR-1 caused post-transcriptional repression of Kir2.1 and Cx43 protein in ischemic myocardium. At the same time, we also observed a downregulation of *KCNJ2* and *GJA1* mRNA both in murine MI models and in miR-1 overexpression. The result indicated that miR-1 might also had effects on the mRNA stability of *KCNJ2* and *GJA1* genes. In concordance with our study, some studies demonstrated that the reduction of *GJA1* or *KCNJ2* mRNA were associated with increased level of inhibitor miR-1 in MI rats [16]. However, some studies reported that there were no significant changes in these mRNA level both in murine model of MI and viral myocarditis, and in miR-1 overexpression [17, 27]. The divergent results might be due to differences in myocardial regions sampled or the time of sampling. Most importantly, we found that t-AUCB suppressed miR-1 overexpression and restored the impaired target mRNA and protein, which further suggested that the inverse relationship between sEHIs and miR-1 expression. Moreover, we previous studies also showed the beneficial effects of sEHIs on the expression of miR-1 and its target arrhythmia-related genes in neonatal cardiac myocytes [24].

### Study limitations

Anesthesia might influence the incidence of arrhythmias in the sham operated mice. Indeed, the incidence of arrhythmia has been shown to decreased in anesthetized compared with conscious rats. However, we were not able to record continuous ECG for longer periods in the conscious, unrestrained mice because of a lack of an implantable telemetry systems. We showed that the up-regulation of Kir2.1 and Cx43 protein might be contribute to the anti-arrhythmia effects of sEHi in ischemic myocardium. However, we did not explore whether other ion channel ion channels proteins (for instance Ca2+ cycling) also involved this process. Previous studies have shown t-AUCB could prevent Ca2+ dysregulation and sarco(endo)plasmic reticulum Ca2+-ATPase (SERCA) remodeling in hyperglycemic rats [28]. Moreover, the proteins involved in the calcium handling could also be regulated by miR-1 [29, 30]. Therefore, further work is warranted to investigate whether the anti-arrhythmic effects of sEHi were also related to Ca2+ cycling in the ischemic myocardium.

In conclusion, sEHIs increase *KCNJ2*/Kir2.1 and *GJA1/Cx43* mRNA/protein by suppressing miR-1 under ischemic arrhythmia conditions. This is predominantly due to increased Akt and GSK-3β phosphorylation.

## MATERIALS AND METHODS

### Materials

miR-1 agomir was purchased from RiboBio (Guangzhou, China). Rabbit anti-mouse *KCNJ2, GJA1,* and β-actin antibodies were purchased from Abcam (Cambridge, UK). TaqMan MicroRNA RT Kit, TaqMan MicroRNA-1a-3p assays, TaqMan U6snRNA assay, and TaqMan Universal PCR Master Mix were purchased from Applied Biosystems (New York, NY, USA).

### Drug delivery

The t-AUCB was a kind gift from Prof. Bruce D. Hammock (University of California, Davis, CA, USA). In a preliminary study, we tested different doses of t-AUCB (5,15, 50 mg/L in drinking-water) in the animal model, and we found that t-AUCB doses above 5 mg/L did not increase the inhibition of miR-1 expression after MI (Supplementary Figure 1A). Therefore, we choose lower concentration gradient of t-AUCB (0.2, 1, 5 mg/L in drinking-water) in our formal test. We added 50 mg t-AUCB to 1000 mL distilled water for make a 50 mg/L stock solution. The samples were sonicated for 1 h until the powder had completely dissolved. The stock solution was diluted to 0.2, 1 and 5 mg/L, and was stored at room temperature. Compared with other sEHIs, t-AUCB has improved water solubility and better oral bioavailability. Therefore, giving t-AUCB in drinking-water is recommended as a feasible and easy route of administration [31, 32]. Mice were observed to drink approximately 6-7 ml water per day which was consistent with other published studies [33], indicate this procedure gives a dose of approximately 0.06-1.4 mg t-AUCB per kg per day. There were no significant differences in the daily water intake between each groups.

### Mice

Animal experiments were in accordance with the NIH Guide for the care and use of Laboratory Animals and approved by the Institutional Animal Care and Use Committee at the Second Xiangya Hospital of Central South University. Thirty-five 8-week-old male Kunming mice (25.03 ± 0.57 g) were randomly divided into five groups (n = 7): (i) sham, (ii) MI, (iii) 0.2 mg/L t-AUCB+MI, (iv) 1 mg/L t-AUCB+MI, (v) 5 mg/L t-AUCB+MI.

### Mouse model of MI

The mice were anesthetized with 1% pentobarbital and underwent left thoracotomy; the trachea was intubated and the mice were ventilated with a small animal respirator (MiniVent Type 845; Hugo Sachs Elektronik, March, Germany). The left anterior descending coronary artery was ligated to create an MI, as described previously [34]. Sham operation was performed without coronary artery occlusion.

### Tissue collection

Mouse hearts were obtained 24h after MI. Ventricular tissues in the border zone of the myocardium of MI mice were dissected and sliced into 2-mm thick. Samples were stored at -80°C.

### Infarct size analysis

The hearts were removed from mice 24h after infarction and kept at -20°C for 1 hour. Frozen ventricles were sliced into 2 mm sections, and the samples were stained with 2,3,5-triphenyltetrazolium chloride (TTC) as previously described [11]. The viable myocardium stained red, and infarct tissues appeared pale white. The area of infarction and left ventricular were measured using Image J. The infarct size expressed as a percentage of the total left ventricular area.

### Immunohistochemistry

Immunohistochemistry was performed as described previously [35]. Briefly, the hearts were fixed with 10% buffered formalin and paraffin embedded, and cut at 4μm section. The left ventricular tissue sections were stained against Kir2.1 and Cx43 (1:300 dilution, Abcam), and treated with the ABC staining system (Santa Cruz Biotechnology). Random fields of view for each of the infarct, border and remote regions were imaged using the microscope system.

### *in vivo* electrophysiologic studies

*In vivo* electrophysiologic studies were performed as previously described [9]. To induce atrial and ventricular tachycardia and fibrillation, programmed extrastimulation techniques and burst pacing were used. For comparison of the inducibility in each mouse, programmed extrastimulation techniques and stimulation duration of atrial and ventricular burst pacing were the same in all mice. Sustained atrial or ventricular arrhythmias were defined as atrial arrhythmias lasting longer than 30 seconds. Reproducibility was defined as greater than one episode of induced atrial or ventricular tachycardia.

### *in vivo* gene transfection

Male Kunming mice were randomly divided into five groups (n = 7): (i) sham, (ii) MI, (iii) agomir+MI, (iv) agomir-NC+MI, (v) agomir+5 mg/L t-AUCB+MI. In a preliminary study, we tested different doses of agomir (10,15,25 nM) and antagomir (20,50,100,200 nM) in the animal model, and we found that the minimal agomir dose (10 nM) were able to increase miR-1 level in MI mice for above 10-fold compared with the control (Supplementary Figure 2), while all doses of antagomir did not affect miR-1 exprssion after MI. Therefore, we focused on the study of agomir treatment in the animal model. Agomir of miR-1 (10 nM of ribonucleotide diluted in 0.2 mL saline) were injected via the tail vein after occlusion. As a control, agomir-negative control (agomir-NC) were injected via the tail vein. Experimental measurements were made 24 h after tail vein injection.

### Real-time PCR detection of miR-1, KCNJ2, and GJA1 mRNA

Total RNA was isolated with TRIzol reagent (Invitrogen) from the mouse hearts obtained post-MI. miR-1 level were analyzed by real-time reverse transcription (RT)-PCR using the TaqMan MicroRNA RT Kit (Applied Biosystems). In brief, 10 ng total RNA was reverse-transcribed with specific stem-loop RT primers using the RT kit according to the manufacturer’s instructions. Real-time RT-PCR was performed on complementary DNA (cDNA) using specific primers designed based on the mouse miR-1 sequence. miR-1 expression was calculated after normalization to U6. Primers for miR-1 were 5′-TCAATCTCTAACAAGCTAATCTCT-3′ (forward) and 5′-TTGACAGTAGGTTAATCCAAAGT-3′ (reverse).

For *KCNJ2* and *GJA1* mRNA quantification, total RNA was extracted with the use of TRIzol. Complementary DNA was prepared with the use of Revert Aid First Strand cDNA synthesis kit (Fermentas). Sybr green quantitative polymerase chain reaction was performed. The quantitative assay was performed using *GAPDH* expression as the internal control. Relative expression was calculated using the comparative threshold cycle (Ct) method (2^-ΔΔCt^). PCR was performed using the following primers:

*KCNJ2:* forward 5′-GGAATGGCAAGAGTAAAGTCCA-3′,

    reverse 5′-AGGGCTATCAACCAAAACACA-3′;

*GJA1*: forward 5′-CTTGGGGTGATGAACAGT-3′,

    reverse 5’-TGAGCCAAGTACAGGAGT-3′;

### Western blotting

Immunoblotting was performed as previously described [36]. The primary antibodies against Kir2.1 (1:1000), Cx43 (1:2000) were purchased from Abcam (Cambridge, MA); Akt and p-Akt (Ser473) (1:2000), GSK-3βand p-GSK-3β(Ser9) (1:2000) were purchased from Cell Signaling Technology (Danvers, MA). GAPDH or β-actin were used as the internal loading control.

### Measurement of the concentrations of EETs in the ischemic myocardium

The levels of EETs and DHETs were measured in the ischemic myocardium. Samples were extracted, seperated by reverse-phase high performance liquid chromatography (HPLC) and analysed by negative-mode electrospray ionization and tandem mass spectrometry (MS) as described previously [37]. It was well recognized that EETs and DHETs were the most biologically active products formed in the cytochrome P450 enzymatic pathway. Therefore, our results were shown as total concentrations of EETs, DHETs and the ratio of EETs to DHETs.

### Statistical analysis

All data are reported as the mean ± SEM. Chi-square analysis was used to compare incidence of arrhythmias in different groups. One-way analysis of variance followed by Bonferroni’s post-hoc test was used for multiple comparisons. All assays were performed in triplicate. A two-tailed *P* < 0.05 was considered significant. Data were analyzed using the SPSS 18.0 statistical package.

## SUPPLEMENTARY MATERIALS FIGURES AND TABLE



## Abbreviations

sEHIs: soluble epoxide hydrolase inhibitors
miR-1: microRNA-1
MI: myocardial infarction
Cx43: encoding connexin 43
EETs: Epoxyeicosatrienoic acids
DHETs: dihydroxyeicosatrienoic acids
βAR: β-adrenoceptor
cAMP: cyclic adenosine monophosphate
PKA: protein Kinase A
SRF: serum response factor
VT: ventricular tachycardia
AVB: atrioventricular block
RMP: resting membrane potential
APD: action potential duration
EAD: early afterdepolarizations
HPLC: high performance liquid chromatography
MS: mass spectrometry
EKG: elecrocardiographic
SERCA: sarco(endo)plasmic reticulum Ca2+-ATPase
t-AUCB: trans-4-[4-(3-adamantan-1-yl-Ureido)-cyclohe-xyloxy]-benzoic acid
