# Supplementary material for: Soluble epoxide hydrolase inhibitors, t-AUCB, regulated microRNA-1 and its target genes in myocardial infarction mice
Source: Oncotarget. 2017 Sep 18;8(55):94635–49. doi: 10.18632/oncotarget.21831 (PMC5706901; doi:10.18632/oncotarget.21831)
Supplement: Supplementary file 1 [file oncotarget-08-94635-s001.pdf]

Soluble epoxide hydrolase inhibitors, t-AUCB, regulated microRNA-1 and its target genes in myocardial infarction mice

SUPPLEMENTARY MATERIALS

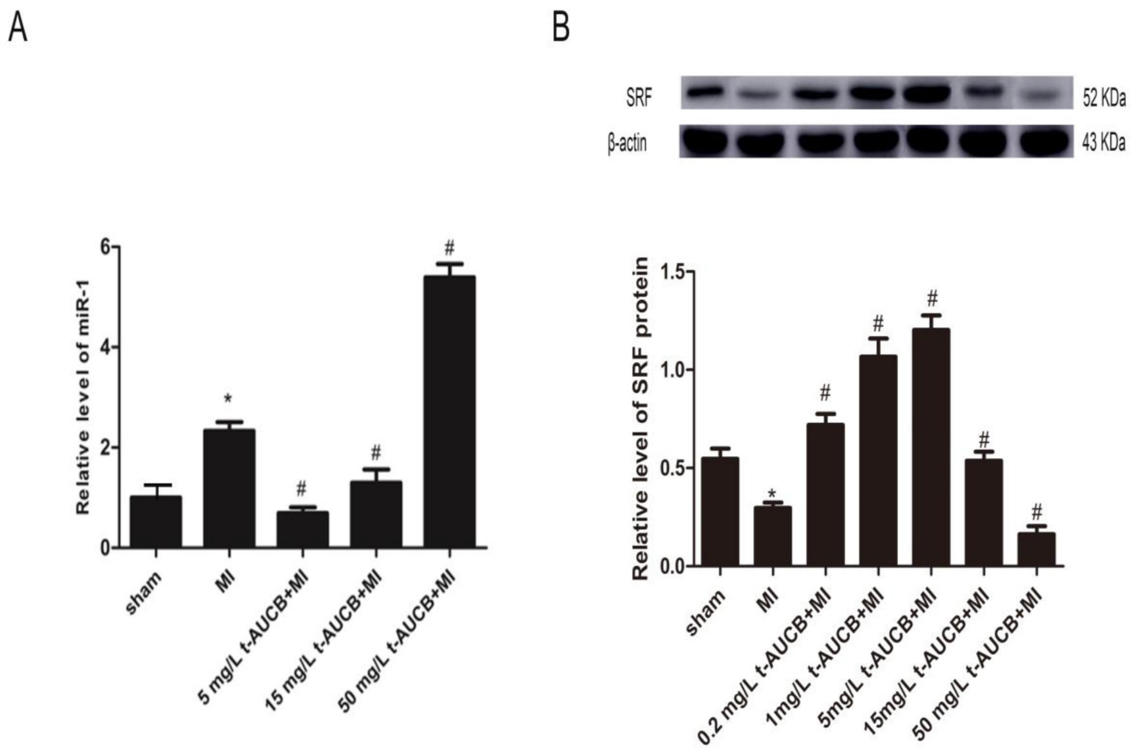

Supplementary Figure 1: Effects of different doses of t-AUCB on expression of miR-1 and SRF protein in ischemic myocardium. Before the MI surgery or sham-operated, mice were randomized to receive either drinking-water or different dose of t-AUCB for seven days. Measurements were made 24h after MI. **(A)** miR-1 was quantificated by real-time PCR. **(B)**. Top, examples of western blot bands; bottom, quantitation as mean ± SEM. *P<0.05 vs. sham group; #P<0.05 vs. MI group. n=3. SRF: serum response factor.

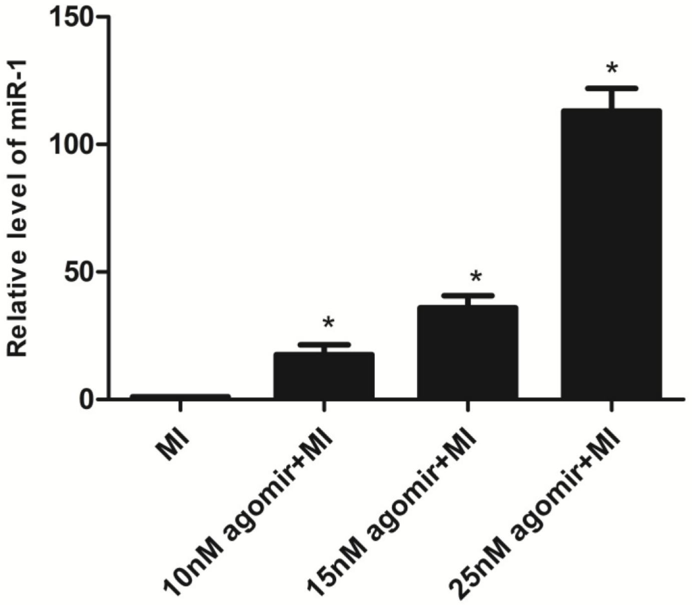

Supplementary Figure 2: Effects of different doses of agomir (10, 15, 25 nM) on expression of miR-1 in ischemic myocardium. The mice were injected with PBS or miR-1 agomir (10, 15, 25 nM) via the tail vein after occlusion. Measurements were made 24h after MI. miR-1 levels were quantificated by real-time PCR. Data were expressed as mean±SEM; *P <0.05 vs. MI group.

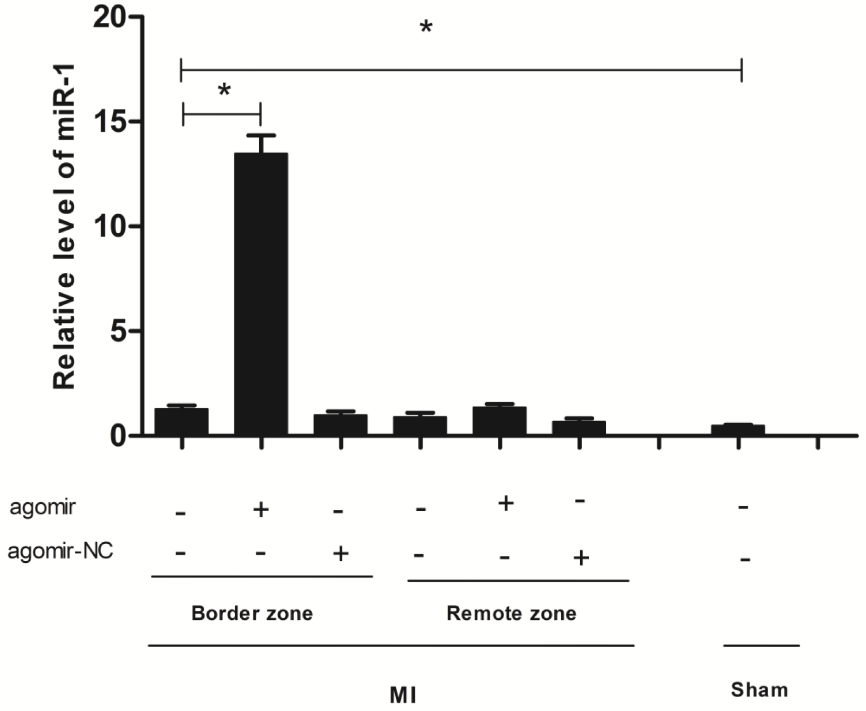

Supplementary Figure 3: Relative levels of miR-1 in mice myocardium after *in vivo* transfer of agomir or agomir-negative control (agomir-NC). Before the MI surgery or sham-operated, mice were randomized to receive either drinking-water or t-AUCB (5 mg/L) for seven days. Mice were transfected with agomir or agomir-NC when had MI surgery. Measurements were made 24h after MI. miR-1 level were quantificated by real-time PCR. All values were normalized to that from the border zone of MI mice.*P<0.05, n=3.

Supplementary Table 1: *In vivo* electrophysiologic studies in MI mice compared with MI mice treated with t-AUCB at 1 week

Treatment	Sham (*n* = 5)	Sham+ t-AUCB (*n* = 5)	MI (*n* =10)	MI+t-AUCB (*n* = 8)	MI+agomir (*n* = 5)	MI+agomir+t-AUCB (*n* =8)
SCL	184 ± 9	172 ± 3	197 ± 11	173±5	202 ± 8	179 ±6
SNRT	228 ± 12	218 ± 6	274 ± 11	221 ±17	258 ± 9	230 ± 10
WCL	92 ±0	92± 0	103 ± 4	96 ± 2	109 ± 3	98 ±2
AVNERP	63 ±0	63 ± 0	79± 3	73± 2	83 ± 2	77 ±3
AERP	46 ± 2	48 ± 0	59 ± 2	52 ±3	71 ± 0	54 ± 2
VERP	37±0	38 ± 2	51 ± 2	46± 2	60± 0	48 ± 3
Atrial arrhythmias	0/5	0/5	3/10*	2/8$^{\#}$	4/5	6/8
Ventricular arrhythmias	0/5	0/5	7/10*	3/8$^{\#}$	5/5	6/8$^{\Delta}$

Data shown represent mean ± SEM. SNRT, sinus node recovery time; WCL, Wenchebach cycle length; AVNERP, AERP and VERP refer to the effective refractory period for the atrioventricular node, atria and ventricles, respectively; AVNERP, AERP, and VERP were performed by using basic cycle length of 120ms; *n* refers to the number of the animals in the studies. **P*<0.05 vs. Sham group; #*P*<0.05 vs. MI group; Δ*P*<0.05 vs agomir +MI group.
